# Characteristics and Dysbiosis of the Gut Microbiome in Renal Transplant Recipients

**DOI:** 10.3390/jcm9020386

**Published:** 2020-02-01

**Authors:** J. Casper Swarte, Rianne M. Douwes, Shixian Hu, Arnau Vich Vila, Michele F. Eisenga, Marco van Londen, António W. Gomes-Neto, Rinse K. Weersma, Hermie J.M. Harmsen, Stephan J.L. Bakker

**Affiliations:** 1 Department of Internal Medicine, Division of Nephrology, University Medical Center Groningen, University of Groningen, 9700RB Groningen, The Netherlands; 2 Department of Gastroenterology and Hepatology, University Medical Center Groningen, University of Groningen, 9700RB Groningen, The Netherlands; 3 Department of Genetics, University Medical Center Groningen, University of Groningen, 9700RB Groningen, The Netherlands; 4 Department of Medical Microbiology, University Medical Center Groningen, University of Groningen, 9700RB Groningen, The Netherlands

**Keywords:** gut microbiome, renal transplant recipient, diarrhea, immunosuppressive medication, gut microbiota, kidney transplantation, 16S rRNA sequencing, butyrate-producing bacteria, Proteobacteria

## Abstract

Renal transplantation is life-changing in many aspects. This includes changes to the gut microbiome likely due to exposure to immunosuppressive drugs and antibiotics. As a consequence, renal transplant recipients (RTRs) might suffer from intestinal dysbiosis. We aimed to investigate the gut microbiome of RTRs and compare it with healthy controls and to identify determinants of the gut microbiome of RTRs. Therefore, RTRs and healthy controls participating in the TransplantLines Biobank and Cohort Study (NCT03272841) were included. We analyzed the gut microbiome using 16S rRNA sequencing and compared the composition of the gut microbiome of RTRs to healthy controls using multivariate association with linear models (MaAsLin). Fecal samples of 139 RTRs (50% male, mean age: 58.3 ± 12.8 years) and 105 healthy controls (57% male, mean age: 59.2 ± 10.6 years) were collected. Median time after transplantation of RTRs was 6.0 (1.5–12.5)years. The microbiome composition of RTRs was significantly different from that of healthy controls, and RTRs had a lower diversity of the gut microbiome (*p* < 0.01). Proton-pump inhibitors, mycophenolate mofetil, and estimated glomerular filtration rate (eGFR) are significant determinants of the gut microbiome of RTRs (*p* < 0.05). Use of mycophenolate mofetil correlated to a lower diversity (*p* < 0.01). Moreover, significant alterations were found in multiple bacterial taxa between RTRs and healthy controls. The gut microbiome of RTRs contained more Proteobacteria and less Actinobacteria, and there was a loss of butyrate-producing bacteria in the gut microbiome of RTRs. By comparing the gut microbiome of RTRs to healthy controls we have shown that RTRs suffer from dysbiosis, a disruption in the balance of the gut microbiome.

## 1. Introduction

It is becoming increasingly evident that the gut microbiome plays a role in various diseases such as inflammatory bowel disease, diabetes, autoimmune diseases, and cancer [1]. However, less is known about the role of the gut microbiome in the field of renal transplantation. Renal transplantation is the best available treatment for patients with end-stage renal disease (ESRD). Despite improved prognosis and quality of life (QoL) compared to dialysis treatment, renal transplant recipients (RTRs) suffer from many problems in the years after transplantation. After transplantation one out of five RTRs suffers from chronic diarrhea which is associated with a lower QoL, increased abdominal complaints, higher mortality, and gut dysbiosis [2,3,4]. Furthermore, all RTRs use immunosuppressive drugs and frequently require antibiotics which potentially influence the gut microbiome [5]. Chronic diarrhea and the use of immunosuppressive drugs may change the gut microbiota composition. As a consequence, this can disrupt gut homeostasis leading to a disruption in the balance of the gut microbiome called dysbiosis. This has previously been reported in mice studies. The introduction of prednisolone and tacrolimus to mice resulted in dysbiosis, an overgrowth of *Escherichia coli*, and an increased colonization with opportunistic pathogens [6]. However, the gut microbiome of RTRs has not been studied extensively.

In previous studies among allogenic stem cell transplant recipients and RTRs, a lower diversity of the gut microbiome was observed [7,8]. Furthermore, this lower diversity of the gut microbiome in allogenic stem cell recipients was associated with a higher risk of mortality [9]. In addition, Annavajhala et al. demonstrated that liver transplant recipients with a lower gut microbiome diversity have a higher risk of colonization by multidrug-resistant bacteria [10]. These studies show that the gut microbiome is clinically relevant in the field of transplantation. However, the role of the gut microbiome in renal transplantation has not been adequately studied. Characterization of the gut microbiome in the first three months after renal transplantation showed significant changes in the composition of the gut microbiome and showed that diarrhea was associated with dysbiosis and a loss of diversity [8]. It is currently unknown whether dysbiosis of the gut microbiome remains prevalent more than one year after transplantation and which factors are determinants of the gut microbiota composition in RTRs. The aim of this study was to characterize the gut microbiome of RTRs for at least more than one year post-transplantation. We compared the composition of the gut microbiome between RTRs and healthy controls and identified determinants of the gut microbiome of RTRs.

## 2. Experimental Section

### 2.1. Study Population

We included 139 RTRs who were at least one year post-transplantation and 105 healthy donors from the TransplantLines Biobank and Cohort Study (ClinicalTrials.gov Identifier NCT03272841). TransplantLines is a prospective observational cohort study in solid transplant recipients [11]. Donors underwent medical screening in the University Medical Center Groningen (UMCG) and can be considered healthy controls. All participants were included during a study visit at the outpatient clinic of the UMCG between September 2015 and April 2018. RTRs were treated with standard antihypertensive and immunosuppressive therapy. The research protocol of the TransplantLines study was approved by the independent medical ethics committee of UMCG (METC 2014/077) and was performed in adherence to the Declaration of Helsinki and the Declaration of Istanbul. All subjects provided a written informed consent.

### 2.2. Patient Characteristics

All measurements were performed during a study visit at the outpatient clinic. Weight, length, and waist and hip circumference were measured in duplicate. Body fat percentage was measured using the multifrequency bioelectrical impedance device (BIA, Quadscan 4000, Bodystat, Douglas, British Isles). Blood pressure was measured by qualified nurses according to a standard clinical protocol as described previously [11]. Hypertension was classified as a mean systolic pressure >140 mm Hg, and/or a mean diastolic pressure >90 mm Hg and/or use of antihypertensive medication. Diabetes mellitus was defined according to the guidelines of the American Diabetes Association [12]. Estimated glomerular filtration rate (eGFR) was calculated using the serum creatinine-based chronic kidney disease epidemiology collaboration (CKD-EPI) formula. Proteinuria was defined as urinary protein excretion >0.5 g per 24 h. Glucose and hemoglobin A1c (HbA1c) were determined using standard laboratory methods. Smoking status was recorded using a questionnaire. Medication use was retrieved from medical records and verified with patients during study visits. The study design is described in detail in the TransplantLines design paper [11].

### 2.3. Sample Collection

Blood samples were collected after an overnight fasting period of 8–12 h and stored at −80 °C. Participants were instructed to collect a fecal sample the day prior to the study visit at home and store the sample on ice. Upon arrival at the UMCG the fecal samples were immediately stored at −80 °C. Participants also collected 24-hour urine samples the day prior to the study visit.

### 2.4. DNA Extraction and 16S rRNA Sequencing

Deoxyribonucleic acid (DNA) was extracted from 0.25 g feces [13]. The genes for the 16S rRNA V4 and V5 region were amplified by polymerase chain reaction (PCR) using the TaKaRa Taq Hot start version kit (TaKaRa Bio Inc., Kusatsu, Japan). We used the 341F and 806R primers containing a 6-nucleotide Illumina-MiSeq adapter sequence. The PCR product was purified with AMPure XP beads (Beckman Coulter, USA). DNA concentrations were measured with Qubit 2.0 Fluorometer to ensure equal library presentation for each sample, dilutions were made accordingly [14]. The normalized DNA library was sequenced using the MiSeq Benchtop Sequencer.

### 2.5. Microbiome Profiling

Bacterial taxonomy was assigned using PAired-eND Assembler for DNA sequences (PANDAseq), Quantitative Insights Into Microbial Ecology (QIIME), and ARB [15,16,17]. QIIME was used to assign taxonomy to the phylum, class, order, family, and genus level. ARB was used to assign taxonomy to the species level. As previously described, PANDAseq was used to increase the quality of sequence reads. Readouts with at quality score lower than 0.9 were discarded according to the protocol followed by Heida et al. [14].

### 2.6. Statistical Analyses

Data are presented as mean ± standard deviation (SD) for normally distributed data and median with interquartile range (IQR) for non-normally distributed data. Differences between baseline characteristics of RTRs and healthy controls were tested using a t-test or a Mann–Whitney u-test.

Sample richness/evenness was estimated using the Shannon index using QIIME. The microbial dissimilarities matrix (Bray–Curtis) was obtained using *vegdist* from the *vegan* R-package [18]. Principal coordinates were constructed and plotted with the *cmdscale* function. We used permutational multivariate analysis of variance using distance matrices (ADONIS) to analyze the variance in the Bray–Curtis matrix that could be explained by metadata such as age, sex, body mass index (BMI), fat percentage, smoking, eGFR, and medication. Pearson correlation was used to correlate metadata to the Shannon diversity index. *p*-values <0.01 were considered statistically significant.

Multivariate analysis by linear models (MaAsLin) is a tool to find associations between clinical metadata and bacterial abundance. We used MaAsLin to find associations between microbiome data and clinical phenotype. MaAsLin performs a boosted, additive general linear model between metadata and microbial abundance [19]. Covariates including sex, body mass index (BMI), smoking, use of antihypertensive medication, use of antibiotics, use of statins, use of proton-pump inhibitor (PPI), and read depth were forced into the model. These covariates are known to influence the gut microbiome [20]. All *p*-values were corrected for multiple testing using false discovery rate (FDR). *p*_FDR_ < 0.10 was considered statistically significant for taxonomic analysis.

## 3. Results

### 3.1. Baseline Characteristics

We included 139 RTRs (age 58.3 ± 12.8 years; 50% males) at a median post-transplantation time of 6.0 (1.5–12.5) years and 105 healthy controls (age 59.2 ± 10.6 years; 57% males). Mean BMI was 27.7 ± 5.4 kg/m² for RTRs and 27.2 ± 6.0 kg/m² for controls. In total 3 (3%) healthy controls and 38 (27%) RTRs had diabetes mellitus (*p* < 0.001). RTRs had a significantly higher HbA1c, 40.0 (37.0–46.0) compared to healthy controls, 37.5 (36.0–40.0) (*p* < 0.001). RTRs had a significantly lower eGFR of 48.3 ± 16.7 mL/min/1.73 m^2^ compared with 69.0 ± 19.2 mL/min/1.73 m^2^ for controls (*p* < 0.001). In total 7 (5%) RTRs used antibiotics, 115 (83%) RTRs used antihypertensive medication, 96 (69%) RTRs used PPIs, and 66 (47%) RTRs used statins. Cyclosporine was used by 25 (18%) RTRs, tacrolimus by 79 (57%) RTRs, azathioprine was used by 13 (9%) RTRs, mycophenolate mofetil by 100 (72%) RTRs, and prednisolone by 133 (96%) RTRs (Table 1).

### 3.2. Diversity of the Gut Microbiome

**Figure 1:**
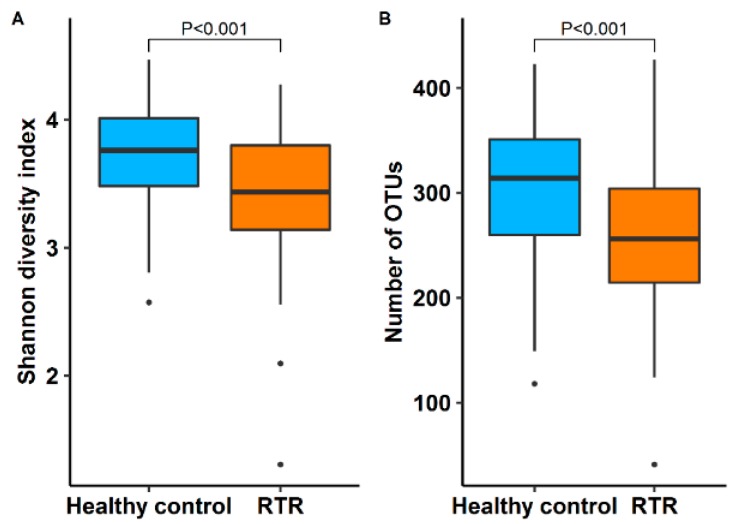
This is a figure showing the diversity of the gut microbiome of renal transplant recipients (RTRs) compared to healthy controls: (**A**) a boxplot depicting the Shannon diversity index, which is a measure for the diversity of the gut microbiome, was significantly lower in RTRs compared to healthy controls (*p* < 0.001); (**B**) a boxplot showing the number of observed operation taxonomic units (OTUs) between RTRs and healthy controls (*p* < 0.001).

**Figure 2:**
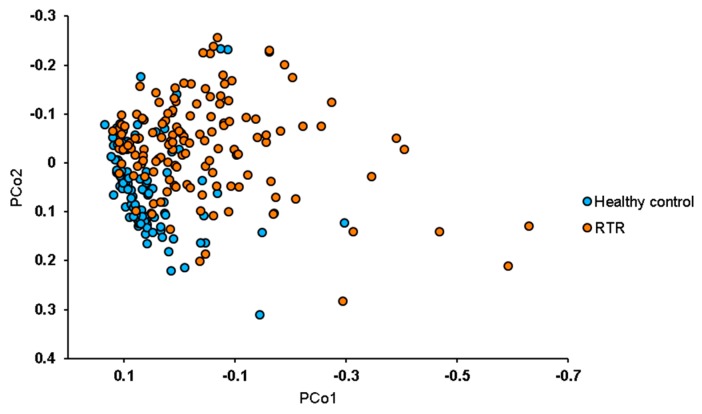
Principal coordinate analysis of 139 RTRs and 105 healthy controls. The principal coordinates plot shows principal coordinates for the Bray–Curtis distance, a measure for the composition of the gut microbiome, for RTRs and healthy controls. Separation in the composition of the gut microbiome between RTRs and healthy controls can be observed. PCo1 is principal coordinate 1 and PCo2 is principal coordinate 2. The gut microbiome of RTRs is significantly different from that of healthy controls in the first coordinate (PCo1 *vs*. PCo2: *p* < 0.01). RTR or healthy control status significantly explained 5.8% of variation in the gut microbiome (*p* < 0.001).

**Figure 3:**
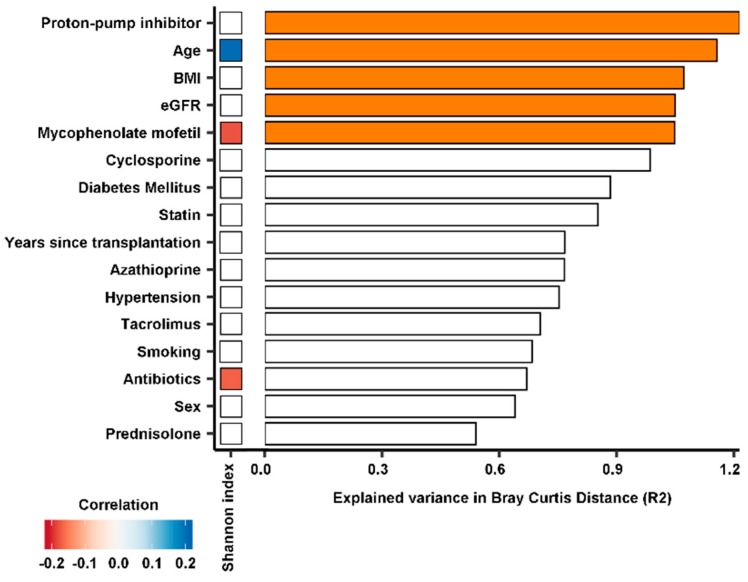
Depiction of variables that are associated with variation in the gut microbiome within RTRs. In the bar plots, the x-axis represents the percentage of explained variance in the gut microbiome of RTRs expressed as the Bray–Curtis distance. The heatmap depicts significant negative correlations (red) and positive correlations (blue) with the Shannon diversity index (*p* < 0.01). These variables were tested only in the gut microbiome of RTRs. Bars in orange represent variables which significantly explain variance in gut microbiota composition (*p* < 0.05).

The median Shannon diversity index, a measure for the diversity of the gut microbiome, was significantly lower in RTR samples with 3.4 (3.1–3.8) *vs*. 3.7 (3.5–4.0) for healthy controls (*p* < 0.001). The median operational taxonomic units (OTUs) per sample was 256 (214–304) for RTRs and 314 (260–351) for healthy controls (*p* < 0.001) (Figure 1). The diversity between samples was further assessed using beta diversity analysis. The gut microbiome was significantly different between RTRs and healthy controls (*p* < 0.01). A separation in gut microbiota composition can be observed between RTRs and healthy controls in the principal coordinate plot (Figure 2). A permutational multivariate analysis of variance using distance matrices (ADONIS) was performed to estimate the variation explained in the gut microbiome by different variables. In total, 5.8% of the variation of the gut microbiome of RTRs and healthy controls was significantly explained by sample type (RTR or healthy control, *p* < 0.001). Furthermore, using ADONIS, baseline characteristics including medication use were tested in the gut microbiome of RTRs. Within the gut microbiome of RTRs age (1.2%), BMI (1.1%), and eGFR (1.0%) significantly explained variation within the gut microbiome. Furthermore, the use of PPIs (1.2%) and the use of mycophenolate mofetil (1.0%) significantly explained variation within the gut microbiome of RTRs. Age was positively correlated to the Shannon diversity index (*p* < 0.01). Use of mycophenolate mofetil and use of antibiotics was negatively correlated to the Shannon diversity index (*p* < 0.01) (Figure 3).

### 3.3. Composition of the Gut Microbiome

**Figure 4:**
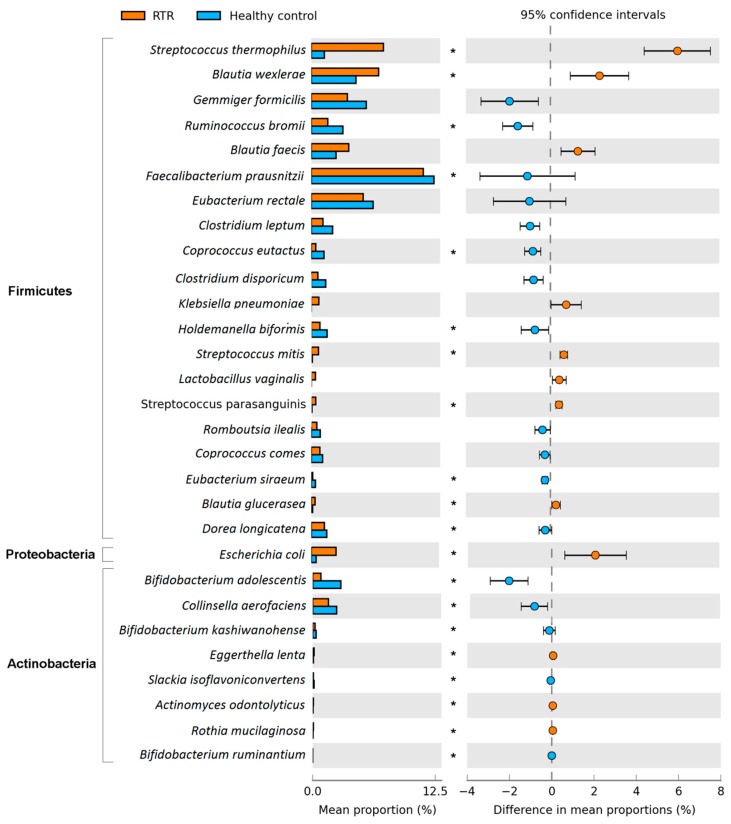
This figure depicts the abundance of phyla and species for RTRs and healthy controls. Bar plots represent the mean proportion and differences in mean proportions with 95% confidence intervals are depicted on the right. Taxa that are depicted were filtered for a difference in mean proportion >0.2%. *p*_FDR_ < 0.10 was considered as statistically significant and indicated in the plot with a star (*).

We analyzed the gut microbiome at different taxonomic levels: phylum, class, order, family, genus, and species. Using MaAsLin, we were able to identify significant differences in taxa abundances between RTRs and healthy controls while correcting for age, sex, BMI, smoking, use of antihypertensive medication, use of antibiotics, use of statins, use of PPIs, and read depth. In total, we found significant alterations in 127 of the 447 bacterial taxa abundances in the gut microbiome of RTRs (*p*_FDR_ < 0.10) (Table 2). On the phylum level we found that RTRs have significantly higher levels of Proteobacteria and lower levels of Actinobacteria (*p*_FDR_ < 0.10) (Figure 4). Within the phylum Proteobacteria, the species *E. coli* was significantly more abundant in the gut microbiome of RTRs (*p*_FDR_ < 0.10). Within the phylum Actinobacteria multiple species had a lower abundance within the gut microbiome of RTRs, especially multiple *Bifidobacterium* species (*p*_FDR_ < 0.10). The predominant phylum Firmicutes was not significantly different in RTRs compared to healthy controls. However, within the phylum Firmicutes there were many significantly different species in the gut microbiome of RTRs compared to healthy controls (Figure 4 and Table S1). An extensive overview of MaAsLin results for complete taxonomy is provided in Table S1.

## 4. Discussion

We have shown that the gut microbiome of RTRs is different compared to the gut microbiome of healthy controls. Interestingly, we demonstrated that RTRs have dysbiosis characterized by general loss of microbial diversity. We found that RTRs have an increased abundance of Proteobacteria, a decrease in Actinobacteria, and a loss of butyrate-producing bacteria. Finally, we found that age, BMI, eGFR, the use of PPIs, and the use of mycophenolate mofetil are determinants of the gut microbiome of RTRs and that age, BMI, and the use of mycophenolate mofetil correlate to the diversity of the gut microbiome.

In a pilot study of Lee et al., significant changes were seen in the gut microbiome of RTRs when pre-transplantation samples were compared to post-transplantation samples. The diversity, although not significant, was lower after transplantation. Proteobacteria and Enterobacteriales were increased in the gut microbiome post-transplantation [8]. We observed a significant loss of diversity in the composition of the gut microbiome in RTRs with a similar increase in Proteobacteria. It is known that a lower diversity is associated with various diseases such as inflammatory bowel disease (IBD), metabolic disease and cardiovascular disease, as stated by the Human Microbiome Consortium [21]. Previous research in a cohort of allogeneic hematopoietic stem cell transplantation (allo-HSCT) recipients demonstrated that a lower diversity of the gut microbiome was associated with a higher mortality risk [9]. Additionally, allo-HSCT recipients who were deceased had a higher level of Gammaproteobacteria, Enterobacteriales, and Enterobacteriaceae [7]. These findings are strikingly similar to the results of our study. We also observed a lower diversity and higher levels of Proteobacteria, Gammaproteobacteria, Enterobacteriaceae, *Escherichia*, *Streptococcus*, and *Lactobacillus* in the gut microbiome of RTRs. However, it is unknown whether these changes in the composition of the gut microbiome are associated with mortality in RTRs.

The lower diversity observed in the gut microbiome of RTRs suggest that RTRs suffer from dysbiosis. We found increased levels of Proteobacteria which has previously been proposed as a marker for dysbiosis in the gut microbiome [22]. Furthermore, we observed a loss of butyrate-producing bacteria in RTRs. Butyrate is a short-chain fatty acid (SCFA) that plays a key role in maintaining gut health. Butyrate is associated with trans-epithelial fluid transport, reduction of inflammation and oxidative stress, reinforcement of the epithelial barrier, and has potential protective properties against colorectal cancer [23]. In this study, lower levels of *Faecalibacterium prausnitzii, Gemmiger formicilis*, *Eubacterium rectale*, *Coprococcus catus*, *Coprococcus comes* and *Roseburia* were observed in the gut microbiome of RTRs. These are all well-known butyrate-producing bacteria [24]. The decrease of butyrate production in RTRs could be detrimental to their gut health. Furthermore, animal studies show that butyrate has immunomodulatory properties through the effect on regulatory T-cells (Treg), which in turn plays a key role in suppressing inflammatory responses. Increasing butyrate in the gut improved renal dysfunction and reduced local and systemic inflammation in mice [25,26]. These results have also been observed in allogeneic bone marrow transplant recipients. Reduced butyrate altered gene regulation and resulted in fewer Treg cells. Restoring butyrate levels led to improved junction integrity, decreased apoptosis, and improved graft versus host disease [27]. Dysbiosis and a loss of butyrate-producing bacteria in the gut microbiome of RTRs could therefore have detrimental effects on gut health. Further research is needed to study the clinical consequences of the loss of butyrate-producing bacteria in RTRs.

In another study of Lee et al., a loss of diversity and a loss of butyrate-producing bacteria was also observed in RTRs with post-transplantation diarrhea. In this study, post-transplantation diarrhea was not associated with common infectious diarrheal pathogens but rather with dysbiosis [4]. RTRs had lower levels of *Ruminococcus*, *Coprococcus*, and *Dorea* in this study. These findings are in accordance with results from our study, which also demonstrated lower levels of *Ruminococcus* in the microbiome of RTRs. However, we did not observe higher levels of *Coprococcus* and *Dorea* in RTRs. One reason for this might be that we included patients more than one year after transplantation while Lee et al. included patients within the first year after transplantation. In addition, we corrected for various factors that influence the composition of the gut microbiome, which was not done in the study by Lee et al. [8].

We found many significant differences in taxa abundance in RTRs compared to healthy controls. Some of these differences in the composition of the gut microbiome might indicate that RTRs suffer from increased inflammation in the gut. For example, the abundance of Proteobacteria was much higher in RTRs compared to healthy controls. This was also observed in patients with severe intestinal inflammation and inflammatory bowel disease (IBD), colorectal cancer, necrotizing enterocolitis, and irritable bowel syndrome (IBS) [28]. Increased oxygen availability in the gut colonocytes could explain these findings, since it is associated with inflammation in the gut and drives the expansion of aerobic Proteobacteria, Enterobacteriaceae, and *E. coli* while lowering the levels of anaerobic bacteria such as *Bifidobacterium* and butyrate-producing bacteria [22,28]. In this study, RTRs indeed had an increased abundance of Proteobacteria, Enterobacteriaceae, and *E. coli*, and lower levels of Clostridia and Bifidobacteria. These similarities in the gut microbiome composition of RTRs and IBD patients suggest that RTRs may be suffering from inflammation in the gut, which could lead to a loss of epithelial barrier function and diarrhea [29].

In this study, we showed that use of PPIs and mycophenolate mofetil was associated with variation within the gut microbiome of RTR. Imhann et al. demonstrated that the use of PPIs changes the gut microbiome, especially an increase of *Streptococcus* species was found in PPI users [30]. We found multiple *Streptococcus* species that had a higher abundance in the gut microbiome of RTRs. This could be due to the use of PPIs. The influence of immunosuppressive medication on the gut microbiome is not yet well studied in RTRs. In a previous murine study, prednisolone, tacrolimus, and mycophenolate mofetil changed the gut microbiome [6]. Mycophenolate mofetil has multiple side effects including diarrhea [31]. In our study, the use of mycophenolate mofetil was significantly correlated to a lower diversity of the gut microbiome. A lower diversity of the gut microbiome is more prevalent in less healthy individuals. More research is needed to investigate the interplay between the use of mycophenolate mofetil, the gut microbiome, and clinical outcomes.

Improving the observed dysbiotic state of RTRs might have clinical implications concerning long-term outcome after renal transplantation. Important issues are that many RTRs suffer from cognitive decline and development of skin cancer [32,33]. Dysbiosis might contribute to cognitive decline due to an effect of the gut microbiome on the gut–brain axis [34]. The same mechanism might apply to the occurrence of skin cancer, due to an effect of the gut microbiome on the gut–skin axis [33,35]. Improving the dysbiotic state of the gut microbiome in RTRs may therefore be a modifiable factor that allows for inhibition of cognitive decline and skin cancer after renal transplantation. Changing the diet of RTRs might be an intervention that allows for improvement of the gut dysbiosis of RTRs. Diet has been identified as an important potentially modifiable factor influencing the gut microbiome [20]. After transplantation, many RTRs adhere to the diet that was prescribed prior to transplantation [36]. This diet includes a protein restriction, which is meant to limit and prevent uremic symptoms and progression of decline of renal function [37]. It also includes a phosphorus restriction to prevent hypophosphatemia, and a potassium restriction to prevent occurrence of hyperkaliemia, cardiac arrhythmias, and acute cardiac death [37]. Improving diet and eating habits may have a positive effect on the composition of the gut microbiome, which could ultimately translate into a beneficial effect on cognitive function [38]. Future, larger cohort studies could focus on the influence of diet on the gut microbiome in RTRs, and on potential sex differences therein. Potential sex differences in the gut microbiome might be of interest, but cohorts to allow for determining those likely need to be large, because previously reported sex differences in the gut microbiome are small [20,39].

We showed that there are many differences in the gut microbiome of RTRs compared to healthy controls. However, the current study should be interpreted within its limitations. The main indication for renal transplantation is chronic kidney disease (CKD). Alterations in the gut microbiome of patients with CKD are already present before transplantation. Patients with CKD also suffer from a lower diversity of the gut microbiome and dysbiosis. A lower colonization by Bifidobacteria and an increase in Enterobacteriaceae was observed in patients with CKD [26]. These findings are similar to our findings which suggest that the dysbiosis observed in RTRs may already be present pre-transplantation and does not recover post-transplantation [8]. Moreover, we measured the composition of the gut microbiome using 16S rRNA sequencing instead of metagenomic sequencing. Therefore, we were unable to analyze metabolic pathways of bacteria. Furthermore, SCFA were not measured in the current study. Therefore, it remains unknown whether the observed loss of butyrate-producing bacteria also leads to a decreased production of butyrate. Future studies should include pre-transplantation patients and study how the gut microbiome develops after transplantation. Focus should be on the metagenome of RTRs to study the effects of dysbiosis, the metabolism genes, and metabolites. Furthermore, current studies of the gut microbiome mainly focus on bacteria. However, at the kingdom level of the gut microbiome, there are many more micro-organisms such as archaea, fungi, eukaryotes, as well as viruses which could also play an important role in the gut microbiome of immunosuppressed patients [40].

In conclusion, the gut microbiome of RTRs more than one year post-transplantation is significantly different from that of healthy controls. The gut microbiome of RTRs contains more Proteobacteria and less Actinobacteria and there is a loss of butyrate-producing bacteria which could be detrimental to gut health. The use of mycophenolate mofetil and antibiotics is associated with variation in the gut microbiome of RTRs and correlated to a lower diversity. The results of this study are preliminary and require replication in a larger cohort. Nevertheless, we demonstrate that RTRs suffer from dysbiosis more than one year post-transplantation and that the use of mycophenolate mofetil correlates to a lower diversity.

## Figures and Tables

**Table 1:** Baseline characteristics of renal transplant recipients (RTRs) and controls.

Demographics	Control	RTRs	*p*-Value
Number of Subjects, *n* (%)	105	139	-
Age (years)	59.2 ± 10.6	58.3 ± 12.8	0.96
Male, *n* (%)	60 (57)	69 (50)	0.24
BMI (kg/m²)	27.2 ± 6.0	27.7 ± 5.4	0.60
Diabetes Mellitus, *n* (%)	3 (3)	38 (27)	<0.001
Hypertension, *n* (%)	10 (10)	115 (83)	<0.001
Smoking, *n* (%)	-	12 (9)	-
Years since Transplantation, Median (IQR)	-	6.0 (1.5–12.5)	-
**Cardiovascular Parameters**			
Glucose, mmol/L, Median (IQR)	5.4 (4.0–5.9)	5.4 (4.9–6.2)	0.06
HbA1c, mmol/L, Median (IQR)	37.5 (36.0–40.0)	40.0 (37.0–46.0)	<0.001
Systolic Blood Pressure (mmHg)	130.4 ± 14.2	136.5 ± 17.7	0.02
Diastolic Blood Pressure (mmHg)	75.8 ± 9.4	78.5 ± 9.6	0.03
Heart Frequency (bpm)	69.7 ± 25.8	72.1 ± 13.1	0.02
**Renal Function Parameters**			
Serum Creatinine (µmol/L)	97.3 ± 22.1	133.1 ± 42.6	<0.001
eGFR (mL/min/1.73 m^2^)	69.0 ± 19.2	48.3 ± 16.7	<0.001
Proteinuria (0.5 g/24 h), *n* (%)	0 (0)	11 (7.9)	-
**Medication, *n* (%)**			
Antibiotics (*n* = 1)	0 (0)	7 (5)	-
Antihypertensive Agents (*n* = 8)	10 (10)	115 (83)	<0.001
Proton-pump Inhibitors	8 (8)	96 (69)	<0.001
Statins	8 (8)	66 (47)	<0.001
Cyclosporine	-	25 (18)	-
Tacrolimus	-	79 (57)	-
Azathioprine	-	13 (9)	-
Mycophenolate mofetil	-	100 (72)	-
Prednisolone	-	133 (96)	-

All characteristics are presented as means ± standard deviation unless otherwise stated. IQR—interquartile range.

**Table 2:** Overview of significantly altered taxa between renal transplant recipients and healthy controls.

Taxonomic Level	Total Number of Taxa ^1^	Number of Significant Taxa ^2^
Phylum	6	2
Class	17	5
Order	31	8
Family	60	18
Genus	123	27
Species	205	63
**Total**	442	123

^1^ Total number of taxa with an abundance >0.1%; ^2^ *p*_FDR_ < 0.10.

## Supplementary Materials

The following are available online at https://www.mdpi.com/2077-0383/9/2/386/s1, Table S1: MaAsLin results for RTRs compared to healthy controls.
